# Surgery of Inverted Papilloma of the Maxillary Sinus via Translacrimal Approach—Long-Term Outcome and Literature Review

**DOI:** 10.3390/jcm8111873

**Published:** 2019-11-05

**Authors:** Tanja Hildenbrand, Rainer Weber, Janina Mertens, Boris A. Stuck, Stephan Hoch, Evangelos Giotakis

**Affiliations:** 1 Department of Oto-Rhino-Laryngology, Medical Center-University of Freiburg, Killianstr. 5, 79106 Freiburg, Germany; tanja.hildenbrand@uniklinik-freiburg.de; 2 Department of Oto-Rhino-Laryngology, Städtisches Klinikum Karlsruhe, Moltkestr. 90, 76133 Karlsruhe, Germany; Janina.Mertens@klinikum-karlsruhe.de; 3 Department of Oto-Rhino-Laryngology, University Hospital Marburg UKGM, Baldingerstraße, 35043 Marburg, Germany; sekretariat.hno.mr@uk-gm.de (B.A.S.); hochs@med.uni-marburg.de (S.H.); 4 Department of Oto-Rhino-Laryngology, 1st ENT University Department of Athens, “Ippokrateio” General Hospital of Athens, Leof. Vasilissis Sofias 114, 11527 Athens, Greece; giotakis@gmail.com

**Keywords:** inverted papilloma, maxillary sinus, prelacrimal approach, endoscopic sinus surgery, endoscopic management, recurrence rate, endoscopic endonasal technique, postoperative symptoms, postoperative morbidity, subperiosteal resection

## Abstract

There are several differential diagnoses of unilateral sinus disease. One of these is inverted papilloma (IP) of the maxillary sinus, which is a common benign tumor with a substantial rate of malignant transformation. In general, endoscopic endonasal techniques for addressing the tumor are favored nowadays instead of classical external approaches. The aim of this retrospective study was to investigate the long-term outcome of inverted papilloma treated endoscopically via the prelacrimal approach. We reviewed 17 patients with primary or recurrent IP of the maxillary sinus that were treated via the prelacrimal endoscopic endonasal technique. After a median follow-up period of 45.9 months (3.8 years), none of the 17 included patients showed signs of recurrent disease and no serious complications were reported. Hypoesthesia of the incisors was reported by four patients and was resolved with time in one. All of the maxillary sinuses could be fully visualized with the flexible endoscope. IP is an important differential diagnosis in the clinical finding of unilateral nasal polypoid lesions. The prelacrimal approach is an effective and safe method in the treatment of IP with limited patient morbidity.

## 1. Introduction

There are several differential diagnoses of unilateral sinus disease with or without polypoid masses in the nasal cavity and middle meatus, e.g. asymmetric manifestation of bilateral chronic rhinosinusitis, odontogenic sinusitis, (antro)choanal polyp, otherwise undefined isolated nasal polyps and benign or malignant tumors [1,2,3,4]. Tumors can mimic inflammatory lesions due to superficial edema hiding the typical endoscopic appearance of such tumors. The most relevant benign tumor is inverted papilloma (IP).

IP of the nose and paranasal sinuses is a benign tumor with a considerable risk for secondary malignancy and a high risk for recurrence [1,2,5,6]. The tumor shows characteristic finger-like invagination of the epithelium in the underlying stroma with aggressive local growth and the tendency to invade the bony surroundings [5,7,8,9]. The treatment of choice is the complete surgical removal of the tumor, which is based on the so-called subperiosteal resection with removal or drilling of the bone at the attachment site [8,10,11,12,13,14,15,16,17,18,19,20,21]. Currently, endoscopic techniques are considered to be the standard procedure for most of the cases [1,2,10,22], but, in principle, an individually adapted surgical strategy is recommended [9,11,12,23,24,25]. Inverted papillomas of the maxillary sinus are difficult to treat, because a considerable portion of the maxillary sinus cannot be adequately visualized and reached, even with the use of angled endoscopes and instruments [10,26,27,28]. Therefore, the classical medial maxillectomy with resection of the medial wall of the maxillary sinus including the inferior turbinate and the nasolacrimal duct or a transoral sublabial access (classic Caldwell-Luc, Canine-fossa-trephination) would be the technique of choice [10,29,30,31,32,33,34,35,36,37,38,39]. However, in external approaches, the problems of visualization and the treatment of regions, like the prelacrimal or alveolar recess remain difficult. Current endoscopic approaches try to preserve the inferior turbinate and nasolacrimal duct, if not involved in the tumor [10,21,40,41,42,43,44,45,46,47,48,49]. The prelacrimal approach to the maxillary sinus is the most recent development. It allows for a complete overview of the maxillary sinus, including the prelacrimal, alveolar and palatine recess and the anterior wall of the maxillary sinus [10,41,42,43,44,45,46]. In combination with angled instruments, it is almost always possible to reach every part of the maxillary sinus [10,50,51,52]. In addition, if the tumor does not affect the inferior turbinate and the nasolacrimal duct, these structures can be preserved [14,40,41,44,45,46,47,48,49,53,54].

Recent publications suggest that the prelacrimal approach further improves the therapeutic outcome with relapse rates of less than 10% and minor postoperative complaints [40,42,43,45,46,49,53]. The aim of this study is to investigate the long-term results (recurrence rate, side-effects, complications) after the endoscopic endonasal resection of inverted papilloma of the maxillary sinus while using the prelacrimal approach, as well as the surgical morbidity.

## 2. Materials and Methods

All patients, operated on by the same surgeon (R.W.) between 2012 and 2016 due to an inverted papilloma of the maxillary sinus via a prelacrimal approach, were screened for inclusion in the study. To be included, patients had to be at least 18 years of age and sign a written informed consent. Informed consent was obtained during the postoperative visit. Patients were given a fact sheet about the study and the use of their personal data in connection with the study according to current European General Data Protection Regulation and they were also personally informed. Patients were excluded if they were less than 18 years old, if informed consent was not given, and they did not have sufficient follow-up data. The study was conducted in accordance with the Declaration of Helsinki and the Guidelines for Good Clinical Practice and the Ethics Committee of the Landesärztekammer Baden-Württemberg, Germany, approved it.

A follow-up of at least 24 months was defined as being reasonable for evaluating long-term results. This was done within the framework of routine check-ups. According to a standardized protocol, the following postoperative symptoms were assessed on a scale of 0–3 (0 = not present, 1 = low, 2 = moderate, 3 = severe): Pain in the upper jaw, nasal stuffiness, increased nasal secretion, or crusting. In addition, it was examined whether a numbness was present in the area of the infraorbital nerve and, in particular, at the incisors, and whether there was any deformation of the alar region on the operated side (0 = no, 1 = yes). Furthermore, we asked for epiphora or recurrent conjunctivitis (0 = no, 1 = yes). If this was reported, a standard dye test (Jones test) (0 ≤ 2 min. transport time, 1 = delayed transport = 2–5 min., 2 = no transport) and passive testing by flushing the lacrimal drainage system with isotonic saline (possible, with increased pressure or not possible) were performed. The nasal cavity and the surgical site were examined by flexible endoscopy. We documented the size of the entrance to the maxillary sinus (>10 mm, 5–10 mm, <5 mm), the visibility of the maxillary sinus (complete or incomplete), the appearance of the mucosa of the maxillary sinus (normal, edematous, presence of purulent secretion), and the presence or absence of recurrence. An MRI was performed to detect a possible mucocele development or tumor recurrence only in those cases where the maxillary sinus could not be completely visualized. The tumor stages were classified according to Krouse [55].

### Surgical Technique

**Figure 1:**
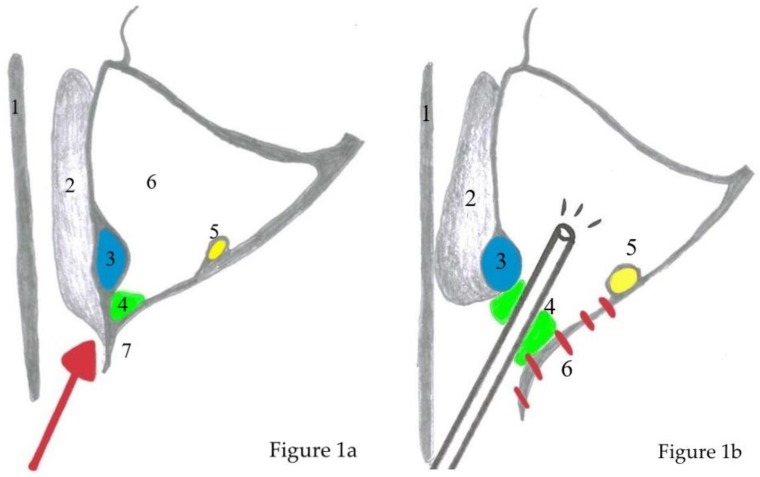
Schematic depiction of anatomy before surgery (**a**) and after prelacrimal approach with an endoscope inside the sinus (**b**). Red arrow = direction of surgery, red lines indicate optional resection of piriform aperture and anterior wall of maxillary sinus. 1 = nasal septum, 2 = inferior turbinate, 3 = nasolacrimal duct, 4 = prelacrimal recess, 5 = infraorbital nerve, 6 = maxillary sinus, 7 = piriform aperture, 8 = endoscope.

**Figure 2:**
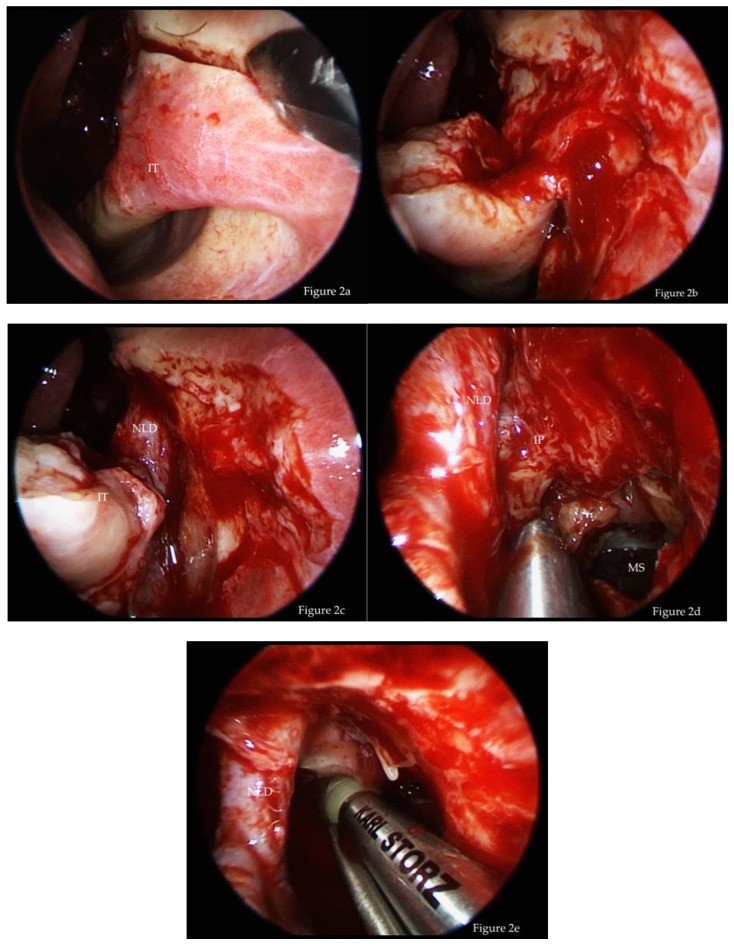
Intraoperative pictures of the prelacrimal approach: (**a**) Horizontal incision at the upper limit of inferior turbinate. (**b**) Dissection of mucosal flaps at the attachment of the inferior turbinate on the lateral nasal wall. (**c**) Exposure of nasolacrimal duct after removal of parts of the frontal process of the maxilla. (**d**) Tumor resection after enlargement of prelacrimal access and medialization of the nasolacrimal duct. (**e**) Drilling of bony attachment of inverted papilloma (IP). IT = inferior turbinate. NLD = nasolacrimal duct. MS = maxillary sinus.

Surgery was performed via HD video endoscopy with 45° and 0° Hopkins endoscopes (KARL STORZ, Tuttlingen, Germany) and a shaver system (Unidrive ENT, KARL STORZ, Tuttlingen, Germany, and Medtronic, Meerbusch, Germany). A CT-scan was obtained for all patients before surgery to examine the tumor expansion, individual anatomy, and possible attachment zones, which are characterized by osteitis and focal hyperostosis with a thickened bone layer [56,57]. The prelacrimal approach was performed as previously described and is briefly summarized (Figure 1a,b, Figure 2a–e) [58].

▪ Key surgical steps are:

- debulking of the exophytic tumor inside the nasal cavity;
- uncinectomy, middle meatal antrostomy type III, with or without opening of the bulla ethmoidalis and an anterior ethmoidectomy;
- entry of the maxillary sinus via the prelacrimal approach with complete exposure of the IP and its attachment;
- creation of a medially based mucosal flap from the nasal floor towards the maxillary sinus;
- subperiosteal resection of the IP and drilling of the bone at the attachment site until the bone shows a clear white color and healthy appearance. In some cases, some parts of the maxillary wall need to be completely resected to the level of the periosteum of the hard palate and the pterygopalatine fossa;
- resection of the nasolacrimal duct if necessary due to tumor invasion;
- performing a medial maxillectomy; and,
- repositioning the mucosal flap and the inferior turbinate, which is sutured to the mucosa of the lateral nasal wall with one or two stitches.

The postoperative care consists of nasal douching with isotonic saline and nasal occlusion for a week. Debridement is performed after the first and second week, depending on the healing process. All the patients were examined every three months during the first year after surgery, every six months during the second year and once a year afterwards. 

## 3. Results

### 3.1. Patient Population

**Figure 3:**
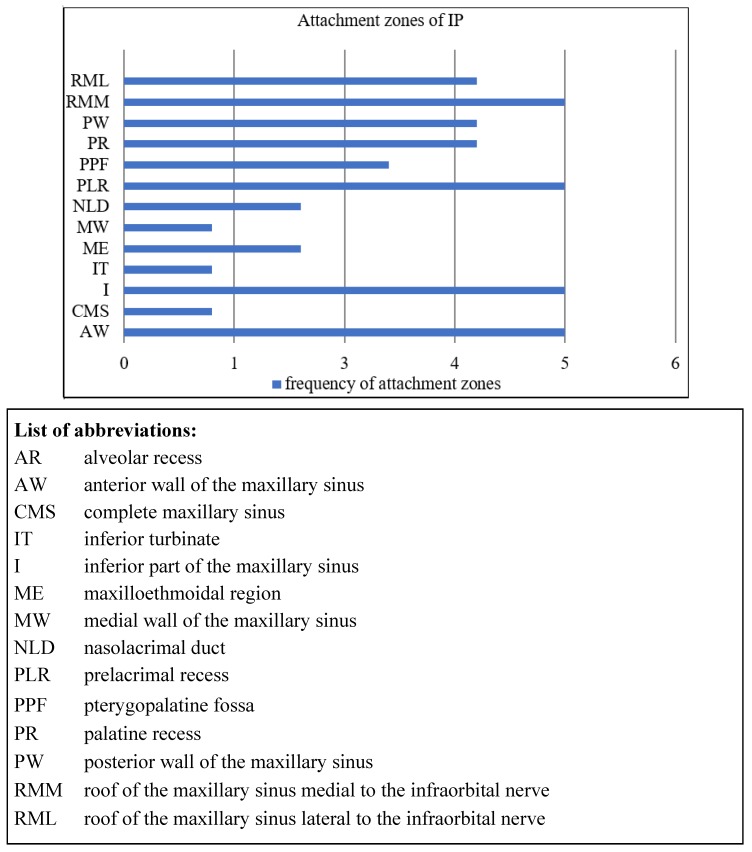
Distribution of IP attachment zones of the maxillary sinus.

A total of 17 Patients, four women and 13 men, with a median age of 54.3 years, were included in the study. Initially, 19 patients with IP of the maxillary sinus were screened for inclusion. One patient changed his phone number and postal address and was not available for follow-up. Another patient with an IP of the maxillary sinus had no prelacrimal approach. A total of 15 patients had a stage III, one patient a stage II, and another patient had a stage IV IP according to Krouse with parts of a carcinoma in situ. Nine patients had recurrent disease. Seven of them had one prior surgery, one patient had two and one patient had three prior surgeries. The median follow-up time was 45.9 months (range 24–69 months). The attachment zones were widely distributed over the maxillary sinus (Figure 3).

### 3.2. Functional Outcome

**Figure 4:**
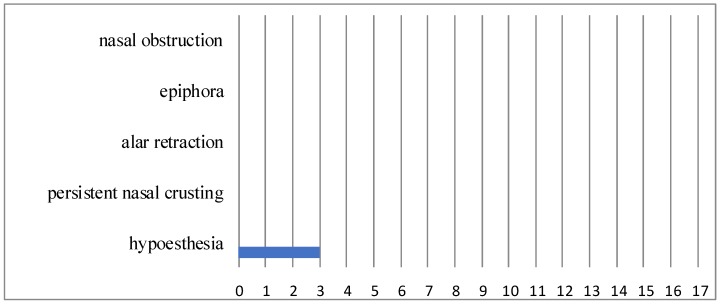
Complaints in long-term evaluation.

The data presented here were collected during the last follow-up visit, at least 24 months postoperatively (median 45.9 months, range 24–69 months). None of the patients had any pain, nasal obstruction, alar retraction, persistent rhinorrhoe, or nasal crusting (n = 0) at the operated side (Figure 4). Four patients complained of hypoesthesia of the incisors immediately after surgery and three of them still complained of hypoesthesia in our long-term evaluation. Another patient had preexisting hypoesthesia of the upper jaw after previous external surgery—this did not change. In two cases, the nasolacrimal duct had to be resected because of tumor infiltration. Both of the patients did not experience epiphora. The inferior turbinate could be preserved in all patients.

### 3.3. Endoscopic Findings

**Figure 5:**
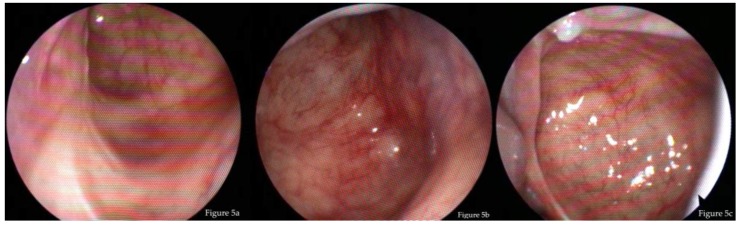
Example of flexible endoscopic inconspicuous findings of a left maxillary sinus after pre-lacrimal approach. (**a**) View of the alveolar recess and posterior lateral wall via inferior nasal meatus; (**b**) View of the anterior wall via inferior nasal meatus; and (**c**) View of the roof, posterior lateral wall, and zygomatic recess via middle meatus

Endoscopically, the opening of the maxillary sinus was wide (>10 mm) in 15 cases (88.2%) and moderate (5–10 mm) in two cases. It was possible to control all parts of the maxillary sinus with the flexible endoscope in all patients, including the anterior wall (Figure 5). 

Four patients showed a volume reduction of the maxillary sinus, and it was unclear whether the size of the maxillary sinus shrank due to scarring and neoosteogenesis, or whether there was some pathologic submucosal process. These four patients were scheduled for further examination by MRI. Additionally, one of them showed increased scarring in the middle meatus. Another patient showed a tumor-like swelling at the inferior turbinate, which turned out to be a fibroepithelial polyp upon histologic examination.

### 3.4. MRI

**Figure 6:**
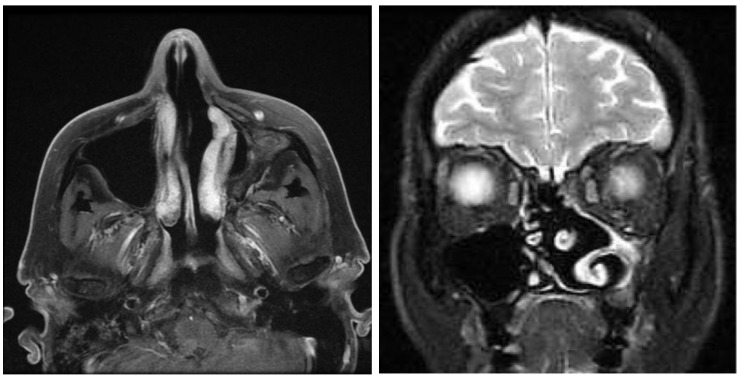
Axial and coronal MRI after prelacrimal approach with a contracted sinus, no signs of recurrence.

**Figure 7:**
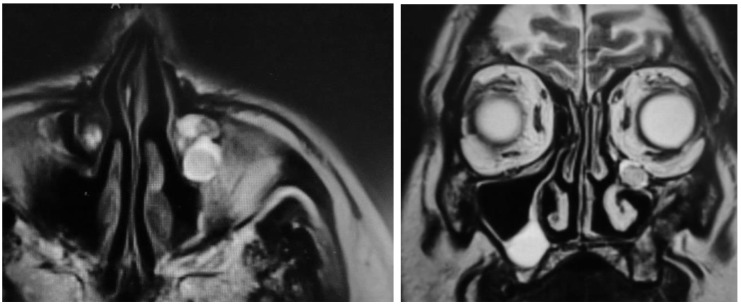
Two mucoceles at the maxillary sinus roof, no signs of recurrence.

One of the four patients refused any radiologic examination due to personal reasons. Endoscopically, he showed a volume-reduced sinus with normal mucosa. None of the three remaining patients showed any tumor recurrence on MRI (Figure 6 and Figure 7). In two patients, small mucoceles were found, which did not cause any symptoms. It was recommended to wait and perform a scan in 1–2 years to control for further growth. In summary, the recurrence rate according to endoscopy and MRI was 0%.

## 4. Discussion

The likelihood of a final diagnosis of a benign or malignant tumor is increased approximately eight-fold in unilateral versus bilateral sinonasal disease [1,4]. IP is found in up to 17% of patients with unilateral sinus disease [2].

### 4.1. Surgical Technique and Recurrence

IP of the nose and paranasal sinuses is treated with endoscopic endonasal techniques in the majority of cases [1,2,22]. These represent the current gold standard approach [1,2,10,22,50]. Reported median recurrence rates are in general between 10%–20% [2,59]. Endoscopic techniques show lower recurrence rates than open approaches (12% vs. 20% [59]; 11.1% vs. 14.2% [2]; 15% vs. 33.3% [60]; 3% vs. 9%–28% [14]; 6%–12% vs. 16% [23]; 7.4% vs. 9% [61]), while older studies present higher recurrence rates than recent studies (12% vs. 20% [59]; 7.7% vs. 11.6% [2]; 13% vs. 14% [23]). This difference could be caused by the use of better surgical techniques, better equipment, and the increasing experience of the surgeons in more recent published studies [2,62]. A meta-analysis of Kim et al. 2017 presented a clear risk reduction for recurrence with endoscopic techniques [22]. The classic medial maxillectomy involves the resection of the whole medial maxillary sinus wall, including the inferior turbinate and the nasolacrimal duct [50,52]. Some authors suggested the temporary dislocation and preservation of the inferior turbinate in order to improve the functional outcome and decrease morbidity [13,40,41,42,43,44,45,46,47,48,49,53,54,63]. The prelacrimal approach provides excellent visibility into the maxillary sinus, including the prelacrimal recess and the anterior wall of the sinus. It also enables the preservation of the inferior turbinate and the nasolacrimal duct [13,38,40,41,42,43,45,46,47,48,49,53,54,63]. If necessary, a wider dissection, involving parts of the piriform aperture and the anterior wall of the maxillary sinus, can be performed [64]. Additionally, if necessary, the infraorbital nerve can be dissected out of its bony canal and preserved to reach attachment zones on the lateral sinus wall, in the case of a protruding infraorbital nerve [65].

Tumors that arise from the lateral or anterior maxillary wall can be thoroughly addressed with 45 or 70 degree endoscopes [10,13,58]. An alternative to the endoscopic procedures is the canine-fossa trephination approach, as part of the transoral approaches [29,31,33,37,39,66,67,68]. The classical Caldwell Luc technique is rarely used nowadays and it is associated with higher recurrence rates [1,14,60,68]. While median recurrence rates of IP in the literature are between 10%–20% [2,59], more recent studies, which present the prelacrimal approach, show significantly lower recurrence rates (1.9% [45]; 0% [49]; 0% [40]). Furthermore, it has been shown that the specific resection technique correlates with recurrence rates. Attachment-oriented resection leads to decreased recurrence rates (<10%) [2,12,16,17,18,21,69,70]. This technique involves subperiosteal tumor resection at the site of tumor origin with a safety margin of 1–1.5 cm [12,15,16,18,38,69]. By using intraoperative negative margins, the recurrence rates can be further reduced [69]. Moreover, there are different recurrence rates, when revision cases are addressed (e.g., 4% vs. 15% recurrence) [60,70,71]. In our study, no revision case showed recurrent disease as of yet. It is still unclear whether revision surgery leads to higher recurrence rates or to the opposite [2]. The expertise of the surgeon might play an important role. When endoscopic techniques do not facilitate adequate tumor exposure or dissection, they should be combined with external approaches [10,30]. This could be due to individual patient anatomy and/or tumor growth and origin. Most recurrences occur in the first two to three postoperative years, but later recurrences are also possible [1,2,5,70]. In our study, we present a median follow-up period of 45.9 months (3.8 years), which is regarded as sufficient for detecting the majority of relapse cases.

### 4.2. Postoperative Morbidity

Surgery-related morbidity after resection of an IP via an endoscopic prelacrimal approach has proven to be low in our study. This is confirmed by the literature [13,40,42,45,47,49]. Persistent nasal crusting, ongoing pain, rhinorrhea, alar retraction, or epiphora have not been observed. The only symptom that has been found in three cases in our long-term examination is hypoesthesia of the upper incisors. In another case, this symptom existed immediately post-surgery, but vanished in the long-term examination. In general, changes of the outer shape of the nose, such as alar retraction, are not frequently described in the literature. Slightly more frequently, epiphora is reported after endoscopic medial maxillectomy, and the most common symptom that is presented in the literature is hypoesthesia of the infraorbital nerve and superior dental plexus [49,72].

The occurrence of hypoesthesia of the upper incisors and the upper jaw is explained by the course of the superior medial alveolar nerve in the mucosa of the anterior maxillary sinus wall. Originating from the infraorbital nerve, it communicates with the superior anterior and posterior alveolar nerve and then forms the superior dental plexus, which innervates these areas [73,74,75,76]. The drilling of the bone or resection of soft tissue in this area consequently leads to sensory deficits. Consequently, this complication cannot be solely ascribed to the surgical technique used or a lack of care of the surgeon, as it would be inevitable due to the individual tumor growth and origin. It is a consequence of necessary tumor resection. Some studies mentioned postoperative crusting and increased nasal secretions, whereas postoperative treatment and nasal care could play a role in the improvement of the above symptoms [15,24,40]. None of the patients that were examined in our study complained of these. There is the assumption that classical EMM leads to a higher risk for lacrimal pathway obstruction and empty nose syndrome [49]. Postoperative nasal care, in general, is rarely mentioned in the cited studies. Suzuki et al. showed, in 2017, that out of 51 examined patients, none had signs of epiphora or changes of the shape of the external nose [45]. Other studies presented epiphora rates of 6% to 13.2% [49,64,72]. Variation in patient cohort, tumor expansion, and surgical technique may cause the difference. Presumably, the risk of nasolacrimal duct obstruction is higher after classical endoscopic medial maxillectomy than after preservation of the nasolacrimal duct. However, if it is infiltrated by the tumor, it needs to be resected to facilitate complete tumor resection as the ultimate goal. Results regarding the occurrence of lacrimal pathway stenosis in the literature are inhomogeneous and hard to compare, as some authors performed dacrycystorhinostomy after medial maxillectomy, some did not and some did it in special cases [15,64,72]. All patients should be informed preoperatively because, overall, paresthesia of the infraorbital nerve is the most common complaint.

Four patients showed a volume reduction of the maxillary sinus. This is a common feature in patients after radical surgery via the Caldwell–Luc approach. It has not been described in the literature in patients after a prelacrimal approach. In a study in rabbits, the extensive removal of mucosa led to a retraction of the medial maxillary sinus wall. Electron microscopy showed inflammation, fibrosis, osteoclastic resorption, increase of osteoclasts within the haversion system, and increased vascularity within the bone [77]. The shrinking of the maxillary sinus is thought to be the result of wound healing. In some patients, the bare bone epithelializes without significant soft tissue reaction and formation. In other patients, the soft tissue and scar tissue formation leads to a reduction of the lumen and a concentric force directed inwards, pulling the bone inwards. There is also osteoneogenesis with a thickening of the bone. Why the wound healing is uneventful in some patients and leads to the mechanism mentioned above in others is currently not known. This does not have any functional impact. Our patients did not report any cosmetic changes of the cheek.

## 5. Limitations

Randomization or blinding were not possible because of the retrospective study design. All of the patients that fulfilled the inclusion criteria were included in the study. Only one patient, who changed his phone number and postal address, was not available for follow-up. Otherwise, the lack of blinding has the advantage that the surgeon himself can examine his patients and knows where the critical points are. The literature of inverted papilloma and surgical techniques cannot be easily compared due to significant variations in tumor size, location, patient cohorts, methods, postoperative treatment, and the number of previous surgeries.

## 6. Conclusions

Inverted papilloma must be considered in patients with unilateral sinus disease and polypoid lesions in the nasal cavity and middle nasal meatus. The endonasal endoscopic prelacrimal approach is a safe technique for the complete removal of inverted papilloma of the maxillary sinus. Postoperative morbidity and complaints are low, without serious complications. Functional results are acceptable. Neither lacrimal duct stenosis, nasal crusting, nor outer shape changes, such as alar retraction, could be observed. The only long-term persistent symptom has been the hypoesthesia of the upper incisors in about 20% of cases, with the tendency to decrease with time. The recurrence rate of 0% after a mean follow-up of 45.9 months (range 12–69 months) reflects the present literature. Consequently, the prelacrimal approach can be used as a standard technique to address maxillary sinus IP. 

## References

[1] Anari S., Carrie S. (2010). Sinonasal inverted papilloma: Narrative review. J. Laryngol. Otol..

[2] Attlmayr B., Derbyshire S.G., Kasbekar A.V., Swift A.C. (2017). Management of inverted papilloma: Review. J. Laryngol. Otol..

[3] Turfe Z., Ahmad A., Peterson E.I., Craig J.R. (2019). Odontogenic sinusitis is a common cause of unilateral sinus disease with maxillary sinus opacification. Int. Forum Allergy Rhinol..

[4] Eckhoff A., Cox D., Luk L., Maidman S., Wise S.K., DelGaudio J.M. (2019). Unilateral versus bilateral sinonasal disease: Considerations in differential diagnosis and workup. Laryngoscope.

[5] Lawson W., Kaufman M.R., Biller H.F. (2003). Treatment outcomes in the management of inverted papilloma: An analysis of 160 cases. Laryngoscope.

[6] Mirza S., Bradley P.J., Acharya A., Stacey M., Jones N.S. (2007). Sinonasal inverted papillomas: Recurrence, and synchronous and metachronous malignancy. J. Laryngol. Otol..

[7] Giotakis E., Eleftheriadou A., Ferekidou E., Kandiloros D., Manolopoulos L., Yiotakis I. (2010). Clinical outcomes of sinonasal inverted papilloma surgery. A retrospective study of 67 cases. B-ENT.

[8] Lund V.J., Howard D., Wei W.I. (2014). Tumors of the Nose, Sinuses and Nasopharynx.

[9] Lund V.J., Stammberger H., Nicolai P., Castelnuovo P., Beal T., Beham A., Bernal-Sprekelsen M., Braun H., Cappabianca P., Carrau R. (2010). European position paper on endoscopic management of tumours of the nose, paranasal sinuses and skull base. Rhinol. Suppl..

[10] Weber R.K., Hosemann W. (2015). Comprehensive review on endonasal endoscopic sinus surgery. GMS Curr. Top. Otorhinolaryngol. Head Neck Surg..

[11] Krouse J.H. (2001). Endoscopic treatment of inverted papilloma: Safety and efficacy. Am. J. Otolaryngol..

[12] Carta F., Blancal J.P., Verillaud B., Tran H., Sauvaget E., Kania R., Herman P. (2013). Surgical management of inverted papilloma: Approaching a new standard for surgery. Head Neck.

[13] Wang C., Han D., Zhang L. (2012). Modified endoscopic maxillary medial sinusotomy for sinonasal inverted papilloma with attachment to the anterior medial wall of maxillary sinus. ORL J. Otorhinolaryngol. Relat. Spec..

[14] Pasquini E., Sciarretta V., Farneti G., Modugno G.C., Ceroni A.R. (2004). Inverted papilloma: Report of 89 cases. Am. J. Otolaryngol..

[15] Eloy P., Mardyla N., Bertrand B., Rombaux P. (2010). Endoscopic endonasal medial maxillectomy: Case series. Indian J. Otolaryngol. Head Neck Surg..

[16] Healy D.Y., Chhabra N., Metson R., Holbrook E.H., Gray S.T. (2016). Surgical risk factors for recurrence of inverted papilloma. Laryngoscope.

[17] Landsberg R., Cavel O., Segev Y., Khafif A., Fliss D.M. (2008). Attachment-oriented endoscopic surgical strategy for sinonasal inverted papilloma. Am. J. Rhinol..

[18] Lombardi D., Tomenzoli D., Butta L., Bizzoni A., Farina D., Sberze F., Karligkiotis A., Castelnuovo P., Nicolai P. (2011). Limitations and complications of endoscopic surgery for treatment for sinonasal inverted papilloma: A reassessment after 212 cases. Head Neck.

[19] Lee T.J., Huang S.F., Huang C.C. (2004). Tailored endoscopic surgery for the treatment of sinonasal inverted papilloma. Head Neck.

[20] Minovi A., Kollert M., Draf W., Bockmuhl U. (2006). [Endonasal micro-endoscopic resection of sinonasal inverted papilloma]. Laryngorhinootologie.

[21] Pagella F., Pusateri A., Giourgos G., Tinelli C., Matti E. (2014). Evolution in the treatment of sinonasal inverted papilloma: Pedicle-oriented endoscopic surgery. Am. J. Rhinol. Allergy.

[22] Kim J.S., Kwon S.H. (2017). Recurrence of sinonasal inverted papilloma following surgical approach: A meta-analysis. Laryngoscope.

[23] Lawson W., Patel Z.M. (2009). The evolution of management for inverted papilloma: An analysis of 200 cases. Otolaryngol. Head Neck Surg..

[24] Zhang L., Li X., Shi L., Cai X., Ye P., Feng X., Pan X. (2014). Endoscopic surgery for maxillary sinus inverted papilloma. Zhonghua Er Bi Yan Hou Tou Jing Wai Ke Za Zhi.

[25] Konstantinidis I., Constantinidis J. (2014). Medial maxillectomy in recalcitrant sinusitis: When, why and how?. Curr. Opin. Otolaryngol. Head Neck Surg..

[26] Robey A., O’Brien E.K., Leopold D.A. (2010). Assessing current technical limitations in the small-hole endoscopic approach to the maxillary sinus. Am. J. Rhinol. Allergy.

[27] Donmez D., Giotakis E., Hosemann W.G., Kuhnel T.S., Hirt B., Weber R.K. (2017). Posterior translacrimal approach to the maxillary sinus. J. Laryngol. Otol..

[28] Hosemann W., Scotti O., Bentzien S. (2003). Evaluation of telescopes and forceps for endoscopic transnasal surgery on the maxillary sinus. Am. J. Rhinol..

[29] Albu S., Baciut M., Opincariu I., Rotaru H., Dinu C. (2011). The canine fossa puncture technique in chronic odontogenic maxillary sinusitis. Am. J. Rhinol. Allergy.

[30] Albu S., Gocea A., Necula S. (2011). Simultaneous inferior and middle meatus antrostomies in the treatment of the severely diseased maxillary sinus. Am. J. Rhinol. Allergy.

[31] Anand V., Santosh S., Aishwarya A. (2008). Canine fossa approaches in endoscopic sinus surgery-our experience. Indian J. Otolaryngol. Head Neck Surg..

[32] Byun J.Y., Lee J.Y., Baek B.J. (2011). Weakness of buccal branch of facial nerve after canine fossa puncture. J. Laryngol. Otol..

[33] Byun J.Y., Lee J.Y. (2013). Canine fossa puncture for severe maxillary disease in unilateral chronic sinusitis with nasal polyp. Laryngoscope.

[34] Bernal-Sprekelsen M., Kalweit H., Welkoborsky H.J. (1991). Discomforts after endoscopy of the maxillary sinus via canine fossa. Rhinology.

[35] Lee J.T., Suh J.D., Carrau R.L., Chu M.W., Chiu A.G. (2017). Endoscopic Denker’s approach for resection of lesions involving the anteroinferior maxillary sinus and infratemporal fossa. Laryngoscope.

[36] Masterson L., Al Gargaz W., Bath A.P. (2010). Endoscopic Caldwell-Luc technique. J. Laryngol. Otol..

[37] Robinson S.R., Baird R., Le T., Wormald P.J. (2005). The incidence of complications after canine fossa puncture performed during endoscopic sinus surgery. Am. J. Rhinol..

[38] Tomenzoli D., Castelnuovo P., Pagella F., Berlucchi M., Pianta L., Delu G., Maroldi R., Nicolai P. (2004). Different endoscopic surgical strategies in the management of inverted papilloma of the sinonasal tract: Experience with 47 patients. Laryngoscope.

[39] Singhal D., Douglas R., Robinson S., Wormald P.J. (2007). The incidence of complications using new landmarks and a modified technique of canine fossa puncture. Am. J. Rhinol..

[40] Erbek S.S., Koycu A., Buyuklu F. (2015). Endoscopic modified medial maxillectomy for treatment of inverted papilloma originating from the maxillary sinus. J. Craniofac. Surg..

[41] Weber R.K., Werner J.A., Hildenbrand T. (2010). Endonasal endoscopic medial maxillectomy with preservation of the inferior turbinate. Am. J. Rhinol. Allergy.

[42] Zhou B., Han D.M., Cui S.J., Huang Q., Wang C.S. (2013). Intranasal endoscopic prelacrimal recess approach to maxillary sinus. Chin. Med. J. (Engl.).

[43] Zhou B., Han D.M., Cui S.J., Huang Q., Wei Y.X., Liu H.C., Liu M. (2007). [Endoscopic nasal lateral wall dissection approach to maxillary sinus]. Zhonghua Er Bi Yan Hou Tou Jing Wai Ke Za Zhi.

[44] Suzuki M., Nakamura Y., Nakayama M., Inagaki A., Murakami S., Takemura K., Yokota M. (2011). Modified transnasal endoscopic medial maxillectomy with medial shift of preserved inferior turbinate and nasolacrimal duct. Laryngoscope.

[45] Suzuki M., Nakamura Y., Yokota M., Ozaki S., Murakami S. (2017). Modified transnasal endoscopic medial maxillectomy through prelacrimal duct approach. Laryngoscope.

[46] Rutherford K.D., Brown S.M. (2010). Endoscopic resection of maxillary sinus inverted papillomas with inferior turbinate preservation. Otolaryngol. Head Neck Surg..

[47] Nakayama T., Asaka D., Okushi T., Yoshikawa M., Moriyama H., Otori N. (2012). Endoscopic medial maxillectomy with preservation of inferior turbinate and nasolacrimal duct. Am. J. Rhinol. Allergy.

[48] Nakamaru Y., Furuta Y., Takagi D., Oridate N., Fukuda S. (2010). Preservation of the nasolacrimal duct during endoscopic medial maxillectomy for sinonasal inverted papilloma. Rhinology.

[49] Pagella F., Pusateri A., Matti E., Avato I., Zaccari D., Emanuelli E., Volo T., Cazzador D., Citraro L., Ricci G. (2017). “TuNa-saving” endoscopic medial maxillectomy: A surgical technique for maxillary inverted papilloma. Eur. Arch. Otorhinolaryngol..

[50] Wormald P.J., Ooi E., van Hasselt C.A., Nair S. (2003). Endoscopic removal of sinonasal inverted papilloma including endoscopic medial maxillectomy. Laryngoscope.

[51] Wormald P.J. (2013). Endoscopic Sinus Surgery: Anatomy, Three-Dimensional Reconstruction and Surgical Technique.

[52] Simmen D.J.N. (2013). Manual of Endoscopic Sinus and Skull Base Surgery.

[53] Morrissey D.K., Wormald P.J., Psaltis A.J. (2016). Prelacrimal approach to the maxillary sinus. Int. Forum Allergy Rhinol..

[54] Gras-Cabrerizo J.R., Massegur-Solench H., Pujol-Olmo A., Montserrat-Gili J.R., Adema-Alcover J.M., Zarraonandia-Andraca I. (2011). Endoscopic medial maxillectomy with preservation of inferior turbinate: How do we do it?. Eur. Arch. Otorhinolaryngol..

[55] Krouse J.H. (2000). Development of a staging system for inverted papilloma. Laryngoscope.

[56] Yousuf K., Wright E.D. (2007). Site of attachment of inverted papilloma predicted by CT findings of osteitis. Am. J. Rhinol..

[57] Sham C.L., King A.D., van Hasselt A., Tong M.C. (2008). The roles and limitations of computed tomography in the preoperative assessment of sinonasal inverted papillomas. Am. J. Rhinol..

[58] Weber R.K., Hosemann W., Kühnel T. (2018). Hands-On Dissection Guide on Advanced Endoscopic Endonasal Sinus Surgery.

[59] Busquets J.M., Hwang P.H. (2006). Endoscopic resection of sinonasal inverted papilloma: A meta-analysis. Otolaryngol. Head Neck Surg..

[60] Kamel R.H., Abdel Fattah A.F., Awad A.G. (2014). Transnasal endoscopic medial maxillectomy in recurrent maxillary sinus inverted papilloma. Rhinology.

[61] Larget I., Bastier P.L., De Gabory L. (2015). External versus endoscopic approach in the management of 131 sinonasal inverted papillomas. Rev. Laryngol. Otol. Rhinol..

[62] Heathcote K.J., Nair S.B. (2009). The impact of modern techniques on the recurrence rate of inverted papilloma treated by endonasal surgery. Rhinology.

[63] Liu Q., Yu H., Minovi A., Wei W., Wang D., Zheng C., Li F., Zhang Z. (2010). Management of maxillary sinus inverted papilloma via transnasal endoscopic anterior and medial maxillectomy. ORL J. Otorhinolaryngol. Relat. Spec..

[64] Dean N.R., Illing E.A., Woodworth B.A. (2015). Endoscopic resection of anterolateral maxillary sinus inverted papillomas. Laryngoscope.

[65] Salzano G., Turri-Zanoni M., Karligkiotis A., Zocchi J., Dell’Aversana Orabona G., Califano L., Battaglia P., Castelnuovo P. (2017). Infraorbital nerve transposition to expand the endoscopic transnasal maxillectomy. Int. Forum Allergy Rhinol..

[66] Sathananthar S., Nagaonkar S., Paleri V., Le T., Robinson S., Wormald P.J. (2005). Canine fossa puncture and clearance of the maxillary sinus for the severely diseased maxillary sinus. Laryngoscope.

[67] Seiberling K., Ooi E., MiinYip J., Wormald P.J. (2009). Canine fossa trephine for the severely diseased maxillary sinus. Am. J. Rhinol. Allergy.

[68] Ghosh A., Pal S., Srivastava A., Saha S. (2015). Modification of endoscopic medial maxillectomy: A novel approach for inverted papilloma of the maxillary sinus. J. Laryngol. Otol..

[69] Miglani A., Hoxworth J.M., Zarka M.A., Lal D. (2018). Use of intraoperative negative margins reduces inverted papilloma recurrence. Am. J. Rhinol. Allergy.

[70] Jiang X.D., Dong Q.Z., Li S.L., Huang T.Q., Zhang N.K. (2017). Endoscopic surgery of a sinonasal inverted papilloma: Surgical strategy, follow-up, and recurrence rate. Am. J. Rhinol. Allergy.

[71] Adriaensen G.F., Lim K.H., Georgalas C., Reinartz S.M., Fokkens W.J. (2016). Challenges in the Management of Inverted Papilloma: A Review of 72 Revision Cases. Laryngoscope.

[72] Bertazzoni G., Accorona R., Schreiber A., Pietrobon G., Karligkiotis A., Fazio E., Castelnuovo P., Nicolai P. (2017). Postoperative long-term morbidity of extended endoscopic maxillectomy for inverted papilloma. Rhinology.

[73] Kasahara N., Morita W., Tanaka R., Hayashi T., Kenmotsu S., Ohshima H. (2016). The Relationships of the Maxillary Sinus with the Superior Alveolar Nerves and Vessels as Demonstrated by Cone-Beam CT Combined With mu-CT and Histological Analyses. Anat. Rec. (Hoboken).

[74] Robinson S., Wormald P.J. (2005). Patterns of innervation of the anterior maxilla: A cadaver study with relevance to canine fossa puncture of the maxillary sinus. Laryngoscope.

[75] Schünke M., Schulte E., Schumacher U. (2009). PROMETHEUS LernAtlas der Anatomie: Kopf, Hals und Neuroanatomie.

[76] Elhadi A.M., Zaidi H.A., Yagmurlu K., Ahmed S., Rhoton A.L., Nakaji P., Preul M.C., Little A.S. (2016). Infraorbital nerve: A surgically relevant landmark for the pterygopalatine fossa, cavernous sinus, and anterolateral skull base in endoscopic transmaxillary approaches. J. Neurosurg..

[77] Moreno P.M., Meseguer D.H. (2008). Bone changes after maxillary sinus surgery: An experimental scanning electron microscopy study. J. Laryngol. Otol..

